# Common Molecular Pathways Between Post-COVID19 Syndrome and Lung Fibrosis: A Scoping Review

**DOI:** 10.3389/fphar.2022.748931

**Published:** 2022-03-04

**Authors:** Laura Bergantini, Alessandro Mainardi, Miriana d’Alessandro, Paolo Cameli, David Bennett, Elena Bargagli, Piersante Sestini

**Affiliations:** Department of Medical Sciences, Surgery and Neurosciences, Respiratory Disease and Lung Transplant Unit, Respiratory Diseases and Transplant Unit, Siena University, Siena, Italy

**Keywords:** lung fibrosis, post covid19 syndrome, molecular aspects, pathogenetic mechanisms, literature review

## Abstract

The pathogenetic mechanism of post-Covid-19 pulmonary fibrosis is currently a topic of intense research interest, but still largely unexplored. The aim of this work was to carry out a systematic exploratory search of the literature (Scoping review) to identify and systematize the main pathogenetic mechanisms that are believed to be involved in this phenomenon, in order to highlight the same molecular aspect of the lung. These aims could be essential in the future for therapeutic management. We identified all primary studies involving in post COVID19 syndrome with pulmonary fibrosis as a primary endpoint by performing data searches in various systematic review databases. Two reviewers independently reviewed all abstracts (398) and full text data. The quality of study has been assess through SANRA protocol. A total of 32 studies involving were included, included the possible involvement of inflammatory cytokines, concerned the renin-angiotensin system, the potential role of galectin-3, epithelial injuries in fibrosis, alveolar type 2 involvement, Neutrophil extracellular traps (NETs) and the others implied other specific aspects (relationship with clinical and mechanical factors, epithelial transition mesenchymal, TGF-β signaling pathway, midkine, caspase and macrophages, genetics). In most cases, these were narrative reviews or letters to the editor, except for 10 articles, which presented original data, albeit sometimes in experimental models. From the development of these researches, progress in the knowledge of the phenomenon and hopefully in its prevention and therapy may originate.

## 1 Introduction

Severe acute respiratory syndrome coronavirus-2 (SARS-CoV-2) infection can generate a systemic disease named coronavirus disease-2019 (COVID-19) (Cameli et al., 2021a).

Since the start of the outbreak, one of the first observations has been the impressive heterogeneity of phenotypic response to SARS-CoV-2 infection among individuals (Benetti et al., 2020). The suitability of antiviral defenses, genetic predisposition together with immunologic defenses is known to influence the severity of the disease and to contribute to the development of COVID-19 associated cytokine storm (Liu et al., 1996; Maltezou et al., 2021; Shakaib et al., 2021). Moreover, sequelae of disease as well as the risk of irreversible organ damage due to COVID-19 are still far from being properly assessed.

Post-COVID syndrome was described for the first time in spring 2020, characterized by persistent symptoms for many weeks after acute infection resolution (Daga et al., 2021; Fallerini et al., 2021; Maltezou et al., 2021). Patients who do not require hospitalization develop post-COVID syndrome in 10–35% cases, while hospitalized patients showed symptoms for up to 80% (Carvalho-Schneider et al., 2021; Cameli et al., 2021b). Even though they are still poorly understood, the immunological dysregulations are probably associated with post-COVID syndrome: in particular, patients with prolonged symptoms duration maintained antigen-specific T-cell response magnitudes to the virus in CD4^+^ and increased T follicular helper cells (Tfh) populations throughout late convalescence while those experiencing a full recover demonstrated an decline of these cellular population (d’Alessandro et al., 2020a; Bergantini et al., 2021a; Files et al., 2021; d’Alessandro et al., 2021b). Different common proteins have been proposed as markers of diagnosis, prognosis or severity for COVID-19 development (d’Alessandro et al., 2021a; Pavli et al., 2021; Billoir et al., 2021), although Post-Acute Sequalae of COVID-19 immune signatures associated with these syndromes are very poorly investigated.

Although review papers are available on post-COVID syndrome (Gianola et al., 2020), to the best of our knowledge, there is no specific evidence concerning the similar molecular aspects between COVID-19 syndrome and chronic, irreversible pulmonary sequelae, such as lung fibrosis. Of note, SARS-CoV2-infection induces direct cytopathic effects against type II pneumocytes, that is considered a key event in the pathogenesis of idiopathic pulmonary fibrosis (IPF) (d’Alessandro et al., 2021a).

The aim of this work was to carry out a systematic exploratory search of the literature (Scoping review) to identify and systematize the main pathogenetic mechanisms that are believed to be involved in this phenomenon, in order to highlight the same molecular aspect of the lung. These aims could be essential in the future for therapeutic management.

## 2 Methods

A scoping review methodology was used, following the methods called scoping review protocol (Tricco et al., 2018). In this paper, descriptive thematic analysis, later detailed, is used to understand any molecular and/or pathways protein involvement present in both IPF and as a consequences of a COVID-19. This article conforms to the Scale for Assessment of Narrative Review Articles (SANRA) guidelines (Baethge et al., 2019).

### 2.1 Eligibility Criteria

The inclusion criteria were peer-reviewed empirical or perspective papers (including editorials or commentaries) with 1) relevance to the study topic: Covid-19 disease or pandemic, fibrogenetic pathways, underlying pathogenetic mechanism, reciprocal influence between SARS-COV-2 and fibrotic lung disease; 2) type of journal: preferences for journals relating to the pneumology area with full text or abstract; 3) type of study: review, case report, case series, original article, letter to the editor. Studies were excluded if 1) did not satisfy the relevance to the topic of study; 2) did not adequately report objectives and conclusions; 3) did not carry full text.

### 2.2 Information Sources and Search

A systematic literature search was conducted from 19 March 2020 to 15 May 2021 in the PubMed, European Centre for Disease Prevention and Control, World Health organization (WHO) Global research on coronavirus disease (COVID-19) (https://www.who.int/emergencies/diseases/novel-coronavirus-2019/global-research-on-novel-coronavirus-2019-ncov), Cochrane Libraryonline database. The search term, which has been included in our Boolean search syntax, is as follows: COVID-19 AND (“lung fibrosis” OR “pulmonary fibrosis” OR “interstitial lung disease”, “pulmonary fibrosis and post-COVID19” OR “pulmonary fibrosis, post- COVID syndrome”). The search was limited to the English language and the availability of the full text and abstracts.

We included elements of the grey literature (e.g., official reports from international organizations), bioXiv, medXiv, arXiv online database. During the initial searches, we have found a living repository of that literature, hosted by the United Nations. That freely accessible repository (https://www.un.org/development/desa/disabilities/covid-19.html (Accessed date: 15 December 2020)) provides key grey literature resources from the United Nations, their specialty agencies, and from partner institutions (e.g., Disabled Person’s Organizations) alike (Management of post-acute, 2021). With this new information, and to produce timely results as intended, we opted to include the grey literature and narrow the review coverage to the peer-reviewed literature and preprint studies. An iterative development process is common in scoping reviews, with some decisions—as long as justified and reported—taken as new information comes by, since scoping reviews usually explore and map out initially unchartered territories (Colquhoun et al., 2020; Jesus et al., 2020; Updated methodological guidance for the conduct of scoping reviews - PubMed, 2021).

### 2.3 Selection Process

The abstract and titles screenings and the full-text assessments were made against the eligibility criteria and were conducted by two independent reviewers (M.d, E.B.), after pilot screenings with over 80% agreements, overseen by the leading review author (L.B.). Any discrepancies were resolved through consensus or the leading author’s input.

### 2.4 Data Charting and Items

**FIGURE 1:**
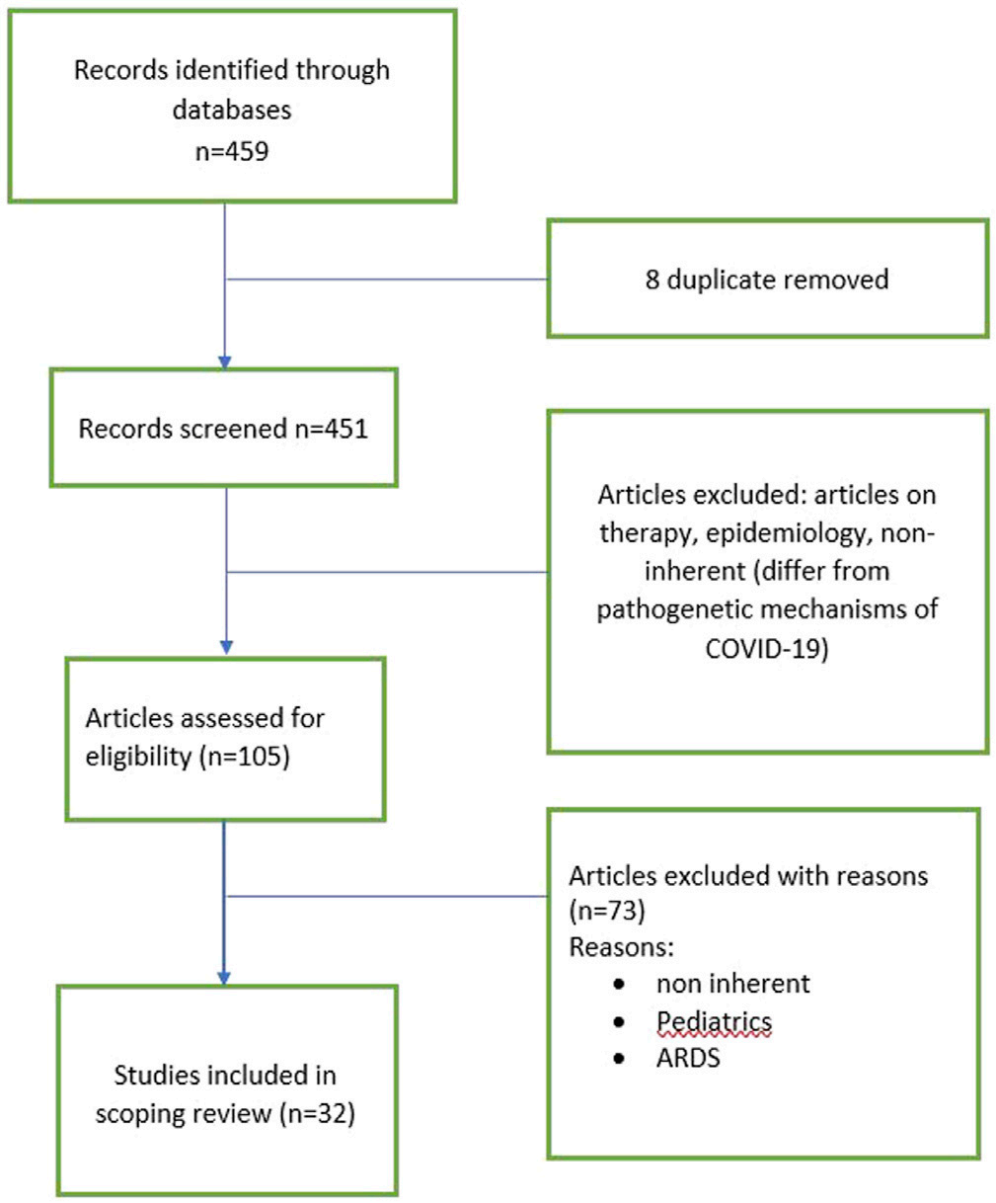
Flowchart of selected manuscript.

Following a coding structure elaborated by members of the research team, one author (D.B.) extracted formal data elements (publication type, sources, geographies addressed, objectives and main findings) with a random 5% verified by another (P.C). Regarding the content of the literature included, three independent reviewers (LB, A.M and P.S.) extracted text quotations on 1) consequences of a COVID-19 infection on people 2) or common molecular pattern with lung fibrosis. These independent extractions were later paired for the qualitative data synthesis, which was also informed by a brief synthesis of each paper developed by two reviewers independently. Then, the content of these extractions after being merged (i.e., presented as the combined extractions of all reviewers), as well as reviewers’ combined synthesis of each paper. Flowchart of selected articles were reported in Figure 1.

## 3 Results

### 3.1 Synthesis of the Results Simple Descriptive Data

**FIGURE 2:**
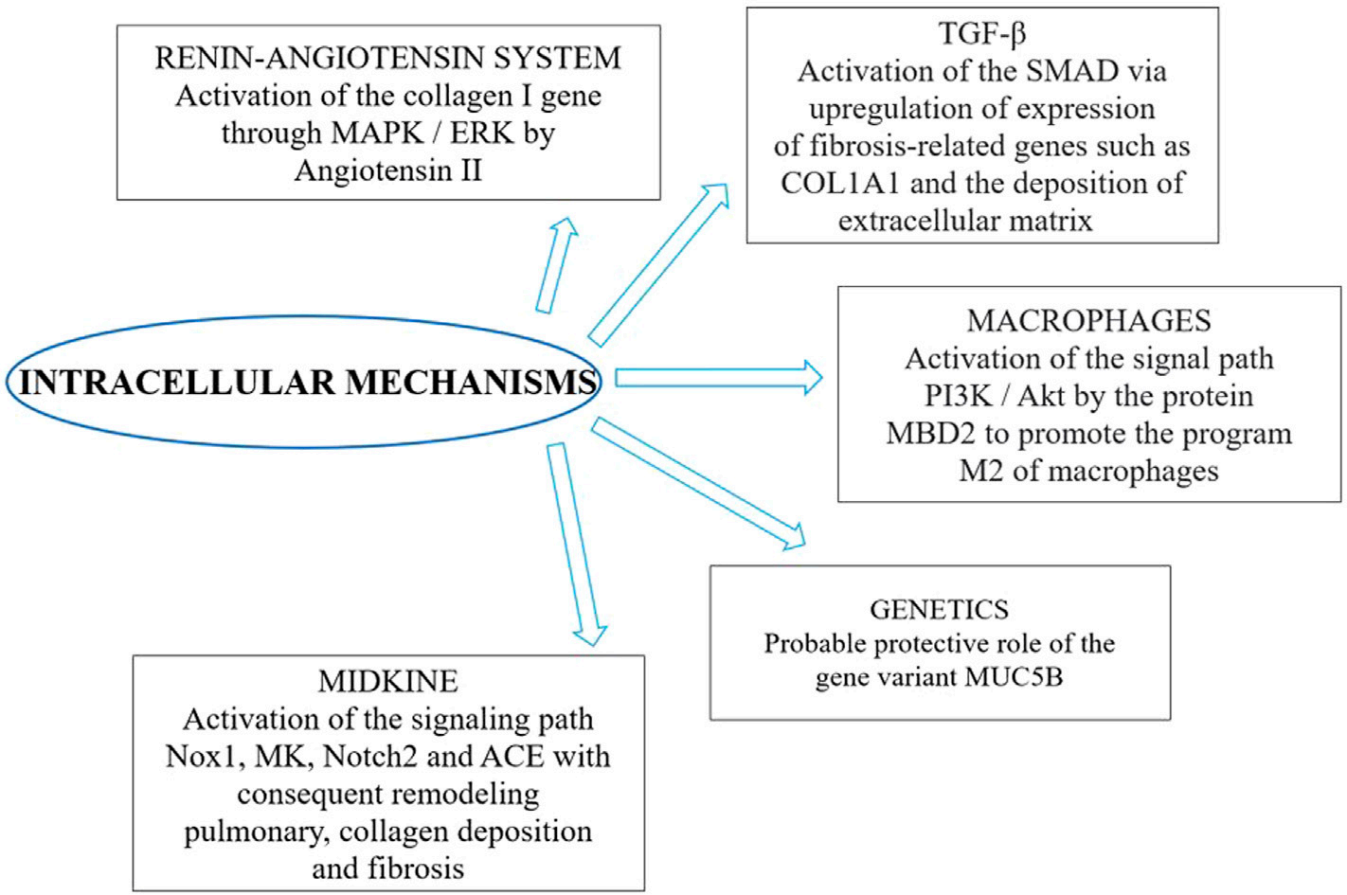
Intracellular mechanisms involved in post COVID-19 syndrome.

**FIGURE 3:**
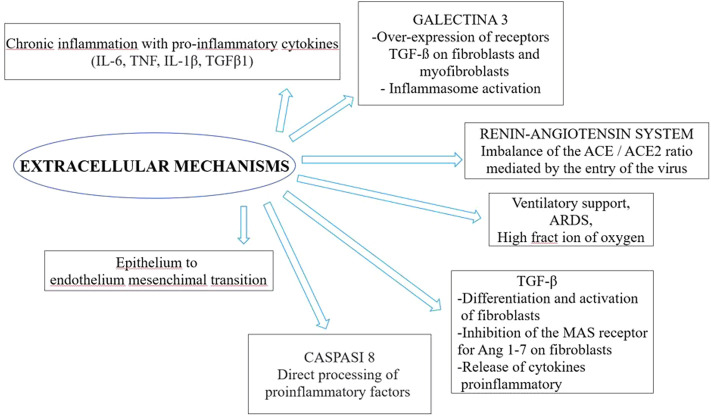
Extracellular mechanisms involved in post COVID-19 syndrome.

**FIGURE 4:**
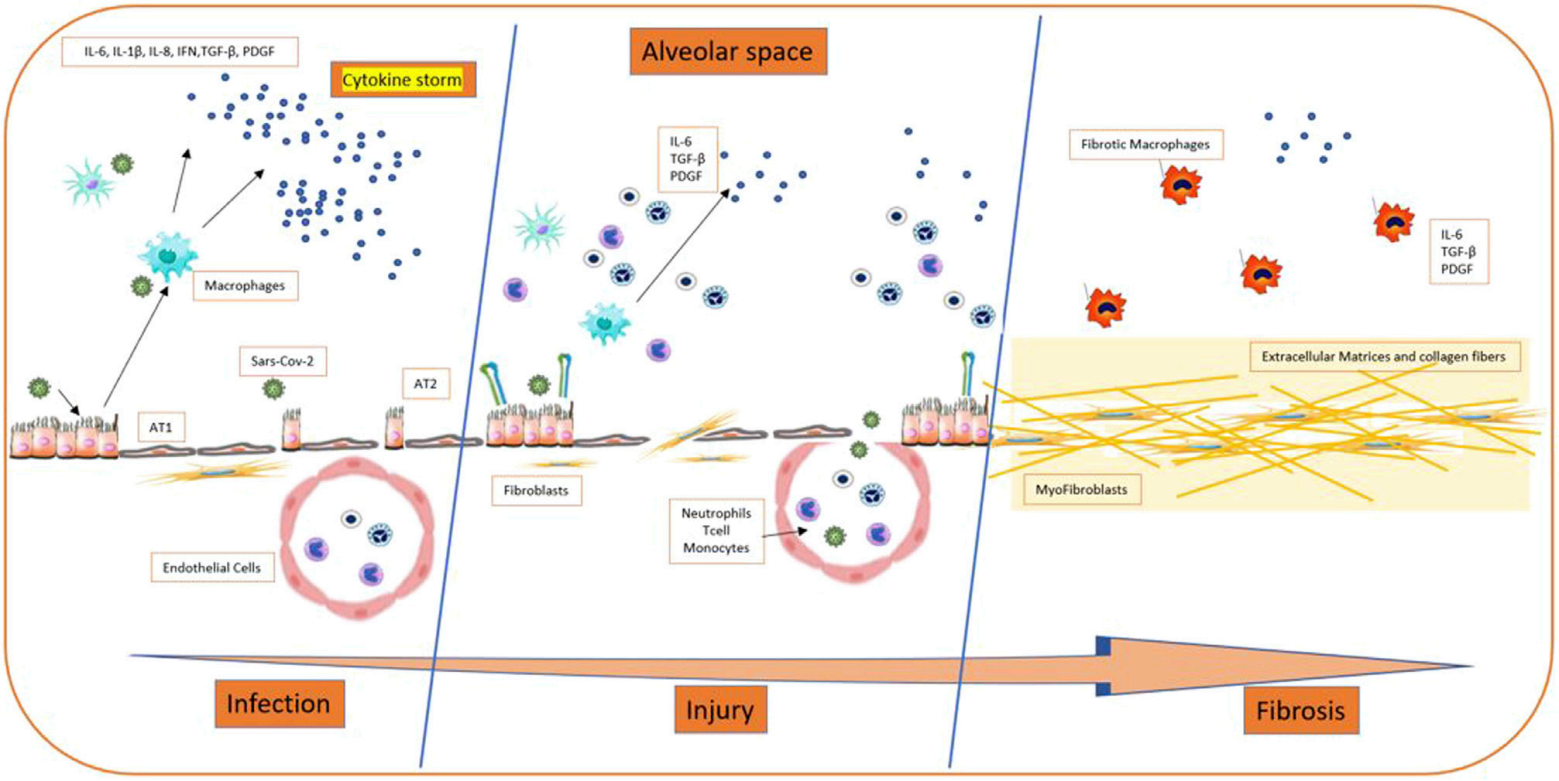
Process of development of fibrosis on Sars COV-2 patients.

The literature search yielded the entire text. Therefore, we selected 32 studies included the possible involvement of inflammatory cytokines, concerned the renin-angiotensin system, the potential role of galectin-3, epithelial injuries in fibrosis, alveolar type 2 involvement, Neutrophil extracellular traps (NETs) and the others implied other specific aspects (relationship with clinical and mechanical factors, epithelial transition mesenchymal, TGF-β signaling pathway, midkine, caspase and macrophages, genetics). These were reviews or letters to the editor, 10 were original article, which presented original data, albeit sometimes in experimental models. The principal extracellular and intracellular mechanisms were reported in Figures 2, 3, while Figure 4 summerized the main pathological processes that lead to fibrosis.

### 3.2 Quality Assessment Following SANRA Assessment

The results of SANRA were reported in Table 1. All 96 ratings (3 raters  ×  32 manuscripts) were used for statistical analysis. The mean sum score across all 32 manuscripts was 8.9 out of 12 possible points (SD 2.6, range 7.75–11, median 9). Highest scores were rated for item 4 (referencing) (mean 1.78), item 6 (Appropriate presentation of data) (mean 1.75) and item 1 (Justification of the article’s importance for the readership) (mean 1.65) whereas items 2, 3, 5 had the lowest scores (means of 1.5, 0.84 and 1.43, respectively).

**TABLE 1:** SANRA Score for quality assessment.

No.	Title and authors	Justification of the article’s importance for the readership	Statement of concrete aims or formulation of questions	Description of the literature search	Referencing	Scientific reasoning	Appropriate presentation of data	Total score
1	Post-COVID lung fibrosis: The tsunami that will follow the earthquake Udwadia ZF, Koul PA, Richeldi L. 2021	1	1	0	2	1	1	6
2	Physiology of Midkine and Its Potential Pathophysiological Role in COVID-19. Sanino G, Bosco M, Terrazzano 2020	1	1	0	2	2	2	8
3	Healing after COVID-19: are survivors at risk for pulmonary fibrosis? McDonald LT. 2021	1	1	0	2	1	2	7
4	A Dangerous Consequence of the Recent Pandemic: Early Lung Fibrosis Following	2	2	1	2	2	2	11
	COVID-19 Pneumonia—Case Reports. Scelfo C, Fontana M, Casalini E, et al., 2020							
5	The second wave of desaturation in coronavirus disease 2019 Alghizzawi MI, Ata F, Yousaf Z, et al., 2021	2	2	1	2	1	2	10
6	Investigation into molecular mechanisms and high-frequency core TCM for pulmonary fibrosis secondary to COVID-19 based on network pharmacology and data mining Yu MX, Song X, Ma XQ, Hao CX, Huang JJ, Yang WH 2021	2	2	2	2	2	2	12
7	Discharge may not be the end of treatment: Pay attention to pulmonary fibrosis caused by severe COVID-19 Zhang C, Wu Z, Li JW, et al., 2021	1	1	0	2	1	2	7
8	MBD2 serves as a viable target against pulmonary fibrosis by inhibiting macrophage M2 program Wang Y, Zhang L, Wu GR, et al., 2021	2	2	2	2	2	2	12
9	Novel insight from the first lung transplant of a COVID-19 patient Chen XJ, Li K, Xu L, et al., 2021	2	2	2	2	1	2	11
10	Post-COVID-19 pneumonia pulmonary fibrosis Tale S, Ghosh S, Meitei SP, Kolli M, Garbhapu AK, Pudi S. 2020	1	0	0	0	1	0	2
11	Persistent viral activity, cytokine storm, and lung fibrosis in a case of severe COVID-19 Xu G, Liu Y, Liao M, et al., 2020	2	2	1	2	1	2	10
12	Lung transplantation for pulmonary fibrosis secondary to severe COVID-19 Bharat A, Querrey M, Markov NS, et al., 2020	2	2	1	2	2	2	11
13	SARS-CoV-2 triggers inflammatory responses and cell death through caspase-8 activation Li S, Zhang Y, Guan Z, et al., 2020	2	1	2	2	2	2	11
14	Endothelial to mesenchymal transition: a precursor to post-COVID-19 interstitial pulmonary fibrosis and vascular obliteration? Eapen MS, Lu W, Gaikwad AV, et al., 2020	1	1	0	2	1	2	7
15	Hyperinflammation and Fibrosis in Severe COVID-19 Patients: Galectin-3, a Target Molecule to Consider Garcia-Revilla J, Deierborg T, Venero JL, Boza-Serrano A. 2020	2	2	0	2	2	2	10
16	SARS-CoV-2 Pathogenesis: Imbalance in the Renin-Angiotensin System Favors Lung Fibrosis Delpino MV, Quarleri J 2020	1	2	0	2	1	2	8
17	Immunopathology of galectin-3: an increasingly promising target in COVID-19 Caniglia JL, Asuthkar S, Tsung AJ, Guda MR, Velpula KK. 2020	2	1	1	2	2	2	10
18	Converging pathways in pulmonary fibrosis and Covid-19 - The fibrotic link to disease severity Wigén J, Löfdahl A, Bjermer L, Elowsson-Rendin L, Westergren-Thorsson G. 2020	1	1	0	2	1	2	7
19	Alveolar cells under mechanical stressed niche: critical contributors to pulmonary fibrosis. Yang J, Pan X, Wang L, Yu G. 2020	2	2	0	2	1	2	9
20	SARS-CoV-2 induces transcriptional signatures in human lung epithelial cells that promote lung fibrosis. Xu J, Xu X, Jiang L, Dua K, Hansbro PM, Liu G. 2020	2	2	2	2	2	2	12
21	Post-COVID-19 Pulmonary Fibrosis: A Lifesaving Challenge Prakash J, Bhattacharya PK, Priye S, Kumar N. 2021	1	1	0	2	1	1	6
22	Long-Term Respiratory and Neurological Sequelae of COVID-19. Wang F, Kream RM, Stefano GB. 2020	2	1	0	2	1	1	7
23	COVID-19: The Potential Treatment of Pulmonary Fibrosis Associated with SARS-CoV-2 Infection Lechowicz K, Drożdżal S, Machaj F, et al., 2020	2	1	0	2	1	2	8
24	Michalski JE, Kurche JS, Schwartz DA. From ARDS to pulmonary fibrosis: the next phase of the COVID-19 pandemic? Transl Res. 2021 September 20:S1931-5244 (21)00243–7. 10.1016/j.trsl.2021.09.001. Epub ahead of print. PMID: 34547499; PMCID: PMC8452088	2	2	1	2	1	1	9
25	Shen H, Zhang N, Liu Y, Yang X, He Y, Li Q, Shen X, Zhu Y, Yang Y. The Interaction Between Pulmonary Fibrosis and COVID-19 and the Application of Related Anti-Fibrotic Drugs. Front Pharmacol. 2022 January 5; 12:805535. 10.3389/fphar.2021.805535. PMID: 35069217; PMCID: PMC8766975	2	1	1	1	2	1	8
26	Stoyanov GS, Yanulova N, Stoev L, Zgurova N, Mihaylova V, Dzhenkov DL, Stoeva M, Stefanova N, Kalchev K, Petkova L. Temporal Patterns of COVID-19-Associated Pulmonary Pathology: An Autopsy Study. Cureus. 2021 December 19; 13 (12):e20522. 10.7759/cureus.20522. PMID: 35103119; PMCID: PMC8769076	1	1	1	1	2	2	8
27	Colarusso C, Maglio A, Terlizzi M, Vitale C, Molino A, Pinto A, Vatrella A, Sorrentino R. Post-COVID-19 Patients Who Develop Lung Fibrotic-like Changes Have Lower Circulating Levels of IFN-β but Higher Levels of IL-1α and TGF-β. Biomedicines. 2021 December 17; 9 (12):1931. 10.3390/biomedicines9121931. PMID: 34944747; PMCID: PMC8698335	2	2	2	2	2	2	12
28	Sinha S, Castillo V, Espinoza CR, Tindle C, Fonseca AG, Dan JM, Katkar GD, Das S, Sahoo D, Ghosh P. COVID-19 lung disease shares driver AT2 cytopathic features with Idiopathic pulmonary fibrosis. bioRxiv (Preprint). 2021 December 26:2021.11.28.470269. 10.1101/2021.11.28.470269. PMID: 34873597; PMCID: PMC8647648	2	2	1	1	2	2	10
29	Giacomelli C, Piccarducci R, Marchetti L, Romei C, Martini C. Pulmonary fibrosis from molecular mechanisms to therapeutic interventions: lessons from post-COVID-19 patients. Biochem Pharmacol. 2021 November; 193:114812. 10.1016/j.bcp.2021.114812. Epub 2021 October 21. PMID: 34687672; PMCID: PMC8546906	2	2	1	1	1	2	9
30	Middleton EA, He XY, Denorme F, Campbell RA, Ng D, Salvatore SP, Mostyka M, Baxter-Stoltzfus A, Borczuk AC, Loda M, Cody MJ, Manne BK, Portier I, Harris ES, Petrey AC, Beswick EJ, Caulin AF, Iovino A, Abegglen LM, Weyrich AS, Rondina MT, Egeblad M, Schiffman JD, Yost CC. Neutrophil extracellular traps contribute to immunothrombosis in COVID-19 acute respiratory distress syndrome. Blood. 2020 September 3; 136 (10):1169–1179. 10.1182/blood.2020007008. PMID: 32597954; PMCID: PMC7472714	2	2	1	1	1	2	9
31	Pandolfi L, Bozzini S, Frangipane V, Percivalle E, De Luigi A, Violatto MB, Lopez G, Gabanti E, Carsana L, D’Amato M, Morosini M, De Amici M, Nebuloni M, Fossali T, Colombo R, Saracino L, Codullo V, Gnecchi M, Bigini P, Baldanti F, Lilleri D, Meloni F. Neutrophil Extracellular Traps Induce the Epithelial-Mesenchymal Transition: Implications in Post-COVID-19 Fibrosis. Front Immunol. 2021 June 14; 12:663303. 10.3389/fimmu.2021.663303. PMID: 34194429; PMCID: PMC8236949	2	2	2	2	2	2	12
32	Mongelli A, Barbi V, Gottardi Zamperla M, Atlante S, Forleo L, Nesta M, Massetti M, Pontecorvi A, Nanni S, Farsetti A, Catalano O, Bussotti M, Dalla Vecchia LA, Bachetti T, Martelli F, La Rovere MT, Gaetano C. Evidence for Biological Age Acceleration and Telomere Shortening in COVID-19 Survivors. Int J Mol Sci. 2021 June 7; 22 (11):6151. 10.3390/ijms22116151. PMID: 34200325; PMCID: PMC8201243	1	1	2	2	1	1	8

### 3.3 Cytokine Pathways and Development of Fibrosis

Most of the studies identified “cytokine storm” as the pathogenetic mechanism leading to fibrosis, based on the release of proinflammatory cytokines. In fact, aberrant inflammation associated with dysregulated repair mechanisms and fibrogenesis can lead to fibrogenesis (Bergantini et al., 2021b; Bergantini et al., 2022). Changes in the cellular and molecular environment in lung tissue secondary to viral infection, as found in COVID-19, were the key factors behind fibrosis development. Ongoing damage occurs in tissues secondary to inflammation, leading to overexpression of inflammatory cytokines, including transforming growth factor-β1 (TGF-β), tumor necrosis factor-α (TNF-α), interleukin-1 (IL-1) and interleukin-6 (IL-6). These mediators, in turn, stimulates the proliferation of type 2 alveolar cells and increases the recruitment of fibroblasts. Eventually, the cascade can lead to an increased production and storage of extracellular matrix (ECM), compromising the architecture of alveolar-capillary membrane leading to impaired gas exchange and hypoxemia (Vietri et al., 2019; Alghizzawi et al., 2021). These assumptions are confirmed by Yu MX, et al., that, by exploiting data mining and network pharmacology, showed that the molecular mechanisms of PF secondary to COVID-19 are mainly related to the TNF signaling pathway, the cytokine-cytokine receptor interaction pathway and the NF-κB signaling pathway. Among cytokines, interleukin 6 (IL-6), TNF and IL-1β have been identified as key targets associated with PF secondary to COVID-19 (Yu et al., 2021).

A recent paper by Colarusso C et al. demonstrated that Post Covid patients with lung fibrosis-like symptoms had higher levels of IL-1α and TGF-β, but lower levels of IFN-β (Colarusso et al., 2021). Interestingly it is well known that IL-1α and TGF-β were highly released by peripheral blood mononuclear cells (PBMCs) obtained by patients with IPF, sharing the same immunological alteration (Terlizzi et al., 2022).

Furthermore, as pointed out by Lechowicz K, et al., there are similar cytokine profiles in IPF and COVID-19, suggesting similar pathological mechanisms underlying fibrosis development between these two diseases. Vesicular-type endothelial cells II are a major source of fibrogenic factors: they stimulate the hyperproliferation of type II follicular cells, recruit fibroblasts in fibrotic loci and induce differentiation and activation of fibroblasts in myofibroblasts. Myofibroblasts are responsible for the excessive accumulation of ECM basement membranes and interstitial tissues, which ultimately leads to the loss of alveolar-capillary barrier function (Lechowicz et al., 2020).

Sinha S. and colleagues with the help of artificial intelligence, demonstrating that COVID-19 resembles idiopathic pulmonary fibrosis (IPF) by sharing prognostic signatures. In particular the pathognomonic Alveolar type 2 (AT2) cytopathic changes, associated with DNA damage induced by oxidative stress that culminates into progenitor state arrest, senescence-associated secretory phenotype (SASP) and an IL15-centric cytokine storm faithfully recapitulate the host immune induced by SARS-CoV-2 and IPF (Sinha et al., 2021). From pathogenetic point of view, in IPF patients AT2 cells was shown to release fibrogenic factors and cytokine (including monocyte chemoattractant protein-1 (MCP-1), TGF-β1, TNF-α, IL-1β, and IL-6). Subsequently, a feedback mechanism stimulates hyperproliferation of AT2 cells, followed by the recruitment of fibroblasts into the fibroblastic foci, and induce the latter to begin myofibroblasts, leading to alveolar function loss (Razzaque and Taguchi, 2003; Satija and Lal, 2007).

### 3.4 The Entrance of the Virus Into the Cells: ACE2 and the Role of Integrins

The renin-angiotensin system (RAS) plays a key role in maintaining blood pressure but is also often involved in lung disease. RAS dysregulation has been implicated in pulmonary fibrosis onset, in particular due to the downstream actions of angiotensin I (Ang I), which is cleaved by ACE into angiotensin II (Ang II); Ang II promotes inflammatory and fibrotic responses through the receptor (AT1R). The other arm in the RAS involves the cleavage of Ang I into Ang (1–9) by (ACE2) and counteracts the ACE arm. Events downstream of Ang (1–9) result in a reduction in inflammation and fibrosis (Gheblawi et al., 2020). In addition, ACE2 also acts on Ang II by converting it into Ang (1–7), which further attenuates inflammation (Wigén et al., 2020). Thus, ACE2 acts as a negative regulator of the renin-angiotensin system. SARS-COV-2 infection is associated with a downregulation ofACE2 expression leading to a ACE/ACE2 ratio imbalance in favor of ACE, thus accelerating the production of angiotensin II (Dalan et al., 2020). The latter, in addition to being a powerful vasoconstrictor, induces the activation of IL-6, TNFα and an increase recruitment of neutrophils and macrophages in alveolar spaces, as well as the direct damage of endothelium. Ang II has also been shown to promote the activation of the collagen I gene via MAPK/ERK in order to generate a fibrotic response (Chang et al., 2021; Giacomelli et al., 2021).

Furthermore, pulmonary fibrosis tissue from IPF patients have shown an increased expression of ACE2 in fibroblasts, make fibrotic patients more susceptible to virus entrance (Shen et al., 2022).

Moreover, SARS-CoV-2 contributes to the activation of the host’s proinflammatory and profibrotic pathways, including those associated with RAS (McDonald, 2021). In support of these hypotheses, the study by Delpino MVet al. points out that higher viral load in respiratory secretions were accompanied by significantly increased serum AngII levels in patients with COVID-19 pneumonia compared to healthy individuals (Delpino and Quarleri, 2020).

Regarding the role of adhesion molecules, it was demonstrated that SARS-CoV-2 virus is able to bind integrins with the consequances of increase viral entry into cells. Αvβ6 Integrin appears to have high affinity for the SARS-CoV-2 virus andit has been directly implicated in IPF pathogenesis, suggesting a link between these seemingly distinct processes (Michalski et al., 2022). In IPF αvβ6 integrin is upregulated and it is able to promote TGFβ1 pro-fibrotic functions. The level of expression correlates with prognosis, making this integrin not only a potential biomarker of disease progression, but also an therapeutic target (John et al., 2020).

### 3.5 Galectin-3 and Pulmonary Fibrosis

The role of Gal-3 as a mediator of pulmonary fibrosis has long been investigated: higher levels of Gal-3 have now been widely associated with the development of interstitial lung diseases. Following cellular stress, the secretion of Gal-3 by macrophages upregulates TGF-ß receptors on fibroblasts and myofibroblasts. This in turn activates these cells, initiating the formation of granulation tissue (*via* collagen deposition) which is eventually remodeled into a fibrous scar. This Gal-3 mediated pathway is widespread throughout the body, not just in the lungs, and is critical for the development of fibrotic change in the liver, kidneys and even the heart (Caniglia et al., 2020). Recently, RNAseq analysis performed on several immune cells in the lungs of COVID-19 patients has provided valuable insights in this setting. Indeed, the study by Garcia-Revilla J, Deierborg T et al. (Garcia-Revilla et al., 2020) points out how Gal3 appears to be elevated in proliferative T lymphocytes associated with severe COVID-19 and that in a subset of pro-fibrogenic macrophages, Gal3 was one of the most upregulated genes in association with TREM2 and SPP1, both of which are involved in the disease and in pulmonary fibrosis. Furthermore, it has also been reported that SARS-CoV-2, like the H5N1 influenza virus, can activate the NLRP3 inflammasome by exploiting the action of Gal3 which in turn governs the release of proinflammatory cytokines such as: IL-1, IL-6, TNFα and IL-1β (d’Alessandro et al., 2020b; Garcia-Revilla et al., 2020; d’Alessandro et al., 2021c).

### 3.6 Other Specific Factors Related to the Development of Lung Fibrosis

#### 3.6.1 Mechanical and Clinical Factors

As mentioned above, it is recognized that the disease caused by SARS-CoV-2 induces the activation of a complex pathway of cytokines that could cause various manifestations lung damage, including a pro-fibrotic pathway (Marchioni et al., 2018). So much so that a similar pro-fibrotic pathway is also involved in the development of some types of pulmonary fibrosis, such as IPF. It should be noted that, in many cases, the clinical syndrome associated with this pathogen is comparable to acute distress syndrome (ARDS), in which fibrotic damage is a possible and well-known consequence.

However there is also significant controversy within the pulmonary community as to the “uniqueness” of COVID-19 ARDS induced pulmonary fibrosis (Bos, 2020).

Mechanical ventilation may also play a role in worsening lung damage and easing the development of fibrosis, and some patients have shown the presence of fibrosis, even after different types of support and varying disease severity. Furthermore, the potential pro-fibrotic role represented by oxygen free radicals (ROS) produced by the prolonged and intensive use of a high-flow oxygen therapy must also be considered (Scelfo et al., 2020).

## 4 Endothelium-Mesenchimal and Epithelium-Mesenchimal Transition

Endothelium-mesenchymal transition occurs when endothelial cells respond to an external insult or internal pathological condition, transforming into a more aggressive mesenchymal state, causing irreversible vascular damage or fibrosis (Yang et al., 2020). Like the endothelium-mesenchymal transition, the epithelium-mesenchymal transition (EMT) is also known to play a crucial role in organ fibrosis. In this process, epithelial cells transform into a mesenchymal phenotype accompanied by basement membrane degradation, epithelial loss and mesenchymal protein gain (Yang et al., 2020). It would appears that this insidious viral infection leads to increased activation of epithelial and endothelial cells through endothelium-epithelial mesenchymal transitions, thus again potentially contributing to post-COVID-19 pulmonary fibrosis (Eapen et al., 2020).

The shared origin of epithelial injury between COVID-19-related ARDS and diseases such as IPF likely represents another unifying aspect of pulmonary fibrosis. Given the connection between the degree of epithelial injury and subsequent fibrosis, these associations could be indicative of a more severe manifestation of post-ARDS fibrosis than other virally-mediated etiologies.

This may also explain why others have described COVID-19-related ARDS as distinct from other types of ARDS (Sisson et al., 2010; Evidence of typepneum, 2022).

Another important mechanisms that highlight similarity between pulmonary fibrosis and COVID-19 regards the role of surfactant proteins. Surfactant is able to form a layer on the alveolar epithelium. At this level, surfactant reduce surface tension that allow the expansion of alveoli and gas exchange (Agassandian and Mallampalli, 2013). Surfactant also participate in host defense against infections and inflammation (Wang et al., 2021a). Alveolar type 2 cells is able to synthesize, secrete, andrecycle pulmonary surfactant, fundamental factor for alveolar stability and host immunity (Calkovska et al., 2021). Moreover it is important to evidence that AT2 produce cytokines and grow factors that affect immunity of the lungs (Calkovska et al., 2021). Surfactant proteins- A, B, C, and D are the main protein of surfanctant.

Several author reported the depletion of surfactant through virus-induced lysis of Type II pneumocytes with associated hyaline membrane formation in COVID-19 ARDS patients (Xu et al., 2020a).

It is also known that polymorphisms of Surfactant Protein (SP)-A, B and D showed association with idiopathic pulmonary fibrosis and various other pulmonary diseases (Wang et al., 2021a).

For these reasons the treatment with surfactant has been proposed for COVID-19 patients (Piva et al., 2021).

## 5 The Roles of TGF-β

TGF-β is a highly expressed key player in nearly all fibrotic processes.

This cytokine promotes redox imbalance by increasing the level of ROS and suppressing antioxidant enzymes. In viral infection-induced pulmonary fibrosis (including SARS-COV-2), oxidative stress increases in epithelial cells, thereby stimulating the production and release of TGF-β, leading to excessive migration, proliferation, activation and differentiation of fibroblasts into myofibroblasts. The latter cells are an important producer of collagenous and non-collagenous matrix molecules. Furthermore, TGF-β regulates AngII-induced collagen expression with subsequent accumulation and inflammation of ECM. In this scenario, activated fibroblasts induce further injury and death in alveolar epithelial cells, thus creating a vicious circle of interactions between profibrotic epithelial cells and fibroblasts that leads to the formation of non-functional scar tissue. Furthermore, TGF-β could also be responsible for inhibiting the expression of the Mas receptor for Ang1-7 in fibroblasts, thus antagonizing the antifibrotic capabilities of the hepatopeptide. In this microenvironment, TGF-β will be able to act on alveolar macrophages by stimulating the secretion of IL-4, IL-6 and IL-13, thus favoring the development of fibrosis (Delpino and Quarleri, 2020). As proof of this, TGF-β messenger RNA transcripts have been observed significantly in alveolar epithelial cells after SARS-CoV-2 infection (Xu et al., 2020b). Furthermore, under stimulation of repeated lesions or inflammation, TGF-β can recruit the type I receptor (TGFB1), thus activating intracellular signaling pathways, in particular the SMAD pathway, with upregulation of the expression of fibrosis-related genes (COL1A1, COL3A1, TIMP1, etc.,) and to the deposition of the extracellular matrix (Vaz de Paula et al., 2021; Zhang et al., 2021). Another interesting thing is that the SARS-CoV-1 nucleocapsid protein can directly promote the upregulation of TGF-β expression. Considering that the similarity of the nucleocapsid protein between SARS-CoV-2 and SARS-CoV-1 is up to 90%, it is possible to hypothesize that SARS-CoV-2 will also have a similar molecular mechanism (Wang et al., 2020). Very recently a study on the autopsy of COVID-19 patients analyzing the morphological and dynamics changes in the pulmonary parenchyma. Type II pneumocyte hyperplasia, alveolar cell multinucleation, and endothelitis resulted the most common alteration observed in this cohort, but without specificity. However the onset of these changes correlates to the onset of clinically established complications and also with the early detection of the development of severe post-COVID syndrome complications (Stoyanov et al., 2021).

## 6 Role of Macrophages

Macrophages are deeply involved in the “dialogue” between innate and adaptive immunity. The function and polarization of macrophages vary greatly depending on their anatomical location and the physical environment in which they are found. Pulmonary macrophages are divided into two subgroups based on their location: alveolar macrophages and interstitial macrophages. In addition to classification by location, macrophages can dynamically move between two activated forms: classically activated (M1) and alternately activated (M2), in response to ever-changing environmental factors. M1 macrophages act primarily as a host defense system to eliminate pathogens by generating pro-inflammatory chemokines and cytokines such as TNF-α, while M2 macrophages exhibit anti-inflammatory properties and engage in remodeling of the extracellular matrix. M2 macrophages are actively involved in the pathogenesis of pulmonary fibrosis (Wang et al., 2020). Furthermore, there is compelling evidence that DNA methylation, one of the main epigenetic mechanisms, is also involved in the pathogenesis of pulmonary fibrosis (Sanders et al., 2012; McErlean et al., 2021). In this regard, there are proteins called MBD, in particular MBD2, which by binding to these methylated areas mediate their activation or repression. Going into more detail, MBD2 improves PI3K/Akt signaling to promote the M2 program of macrophages and consequently the fibrogenesis process. All this is extremely important because MBD2 was found to be highly expressed in pulmonary macrophages of patients with COVID19 and that the loss of MBD2 leads to a marked reduction in the accumulation of M2 macrophages in the lung, with a reduction in fibrotic commitment (Wang et al., 2021b; McErlean et al., 2021).

### 6.1 Role of Caspasi 8

Caspase 8 is the main regulator of several cell death pathways, including apoptosis, necroptosis and pyroptosis. Its role in regulating inflammatory responses has recently been reported in the context of fungal infection (Gringhuis et al., 2012). It would appear that SARS-CoV-2 induces activation of caspase-8 to trigger cell apoptosis and to directly process inflammatory factors such as pro-IL-1β, which are essential later to trigger the pulmonary fibrotic process. Consistent with this notion, massive infiltrations of inflammatory cells, necrotic cell debris, and interstitial fibrosis have been observed in the post-mortem lungs of COVID-19 patients. Overall, these mechanisms could lead to severe lung damage and immune pathogenesis during SARS-CoV-2 infection. Thus, this suggests that caspase-8 activation may play a central role in SARS-CoV-2-induced apoptosis and inflammatory and fibrotic responses (Li et al., 2020).

### 6.2 Role of Midkine and NETosis

Midkine is a heparin-binding growth factor and shows a physiological role in embryonic development (Kadomatsu et al., 1988). It is also poorly expressed in the cells of the adult organism, while it is strongly increased in tumor cells and correlated with a less favorable prognosis in cancer patients (Maeda et al., 2007). As demonstrated by the study by Weckbach et al. it should be noted that midkine is significantly involved in causing inflammation and in the production of proinflammatory cytokines (Weckbach et al., 2011). This growth factor is also an important physiological mediator of RAS and has been found to be significantly increased in patients with ARDS (Kadomatsu, 2010; Zhang and Baker, 2017). Furthermore, midkine, in viral infections, including COVID-19, may promote the infiltration of neutrophils, the formation of NETs and be involved in lung remodeling and fibrosis, through the deposition of collagen through Nox1, MK, Notch2 signaling pathway.

Different factors inducing NETs resulted increased in autopsies of COVID-19 patients (Middleton et al., 2020). In fact NETs promoting cytokine storm through NFκB pathway activation in alveolar epithelial cells that triggers ROS production. Furthermore, NETs can induce the EMT in lung epithelial cells, thus further supporting NET role in fibrosis pathogenesis (Middleton et al., 2020; Pandolfi et al., 2021; d’Alessandro et al., 2022). So much so that anti-midkine monoclonal antibodies have been proposed as a new potential therapeutic strategies in COVID-19 (Sanino et al., 2020).

### 6.3 Genetic Aspects

From a genetic point of view, not much evidence is available in the literature: most studies try to investigate the genetic correlations between IPF and severe COVID-19. In this regard, in one of these studies, it was observed that the most important genetic risk factor for IPF, the variant MUC5B, seems to confer protection against COVID-19. However, it should be noted that the observed effect could be due to the protective effects of mucin overproduction on the airways or be a consequence of the selection bias. Therefore, further investigation is needed to address this apparent paradox (Fadista et al., 2021). Furthermore, in another study based on experimental models, it was found that SARS-CoV-2 infection increases the messenger RNA transcripts of ACE2, TGFB1, CTGF and FN1 in alveolar epithelial cells. These same changes were also found in the lung tissues of patients with pulmonary fibrosis (Xu et al., 2020b).

The epigenetic alteration has also been suggested in the pathogenesis of post-Covid patients developed pulmonary fibrosis. In COVID-19, patients bearing shorter telomeres in their peripheral leukocytes have been proposed to be at risk of worse prognoses. Telomere length is a marker of aging: progressive telomere shortening is a well-characterized phenomenon observed in older adults and attributed to the so-called telomere attrition (Mongelli et al., 2021). Telomere shortening is a strong predictive factor of poor prognosis in patients with IPF and short telomeres have more rapid disease progression (Molina-Molina, 2019; Mongelli et al., 2021).

## 7 Conclusion

In general, fibrosis is a normal repair process and is almost inevitably preceded by other tissue changes and inflammatory reactions. In the case of repeated or chronic lung damage, fibrosis becomes aberrant wound healing due to the dysregulation of the fibroblasts and the extensive deposition of collagen and elastin. While pulmonary fibrosis development and progression is the main actor of IPF, fibrogenic processes are also present in the evolution of COVID-19.

The topic of the pathogenetic mechanism of post-Covid-19 pulmonary fibrosis is currently a topic of intense research interest, but still largely at a speculative level. From the development of these researches, progress in the knowledge of the phenomenon and hopefully in its prevention and therapy may originate.

## Abbreviations

HRCT, High Resolution Computed Tomography; IL-, interleukin; IPF, Idiopathic pulmonary fibrosis; LFT, Lung Function Tests; PFT, Pulmonary Functional Test; TGF-β, Tumor Growth Factor-β.
